# Necroptosis: Molecular Signalling and Translational Implications

**DOI:** 10.1155/2014/490275

**Published:** 2014-01-23

**Authors:** Claudia Giampietri, Donatella Starace, Simonetta Petrungaro, Antonio Filippini, Elio Ziparo

**Affiliations:** Department of Anatomical, Histological, Forensic and Orthopaedic Sciences, Section of Histology and Medical Embryology, Sapienza University of Rome, Via Scarpa 14, 00161 Rome, Italy

## Abstract

Necroptosis is a form of programmed necrosis whose molecular players are partially shared with apoptotic cell death. Here we summarize what is known about molecular signalling of necroptosis, particularly focusing on fine tuning of FLIP and IAP proteins in the apoptosis/necroptosis balance. We also emphasize necroptosis involvement in physiological and pathological conditions, particularly in the regulation of immune homeostasis.

## 1. Introduction

In 1998 Vandenabeele's group demonstrated that murine L929 fibrosarcoma cells treated with the pan-caspase inhibitor zVAD-FMK rapidly die in a necrotic way after tumor necrosis factor (TNF) incubation, indicating a possible involvement of caspases in protection against TNF-induced necrosis [1]. Additional works then described this particular form of cell death having many hallmarks of cellular necrosis and induced by death receptor stimulation [2, 3]. Further studies performed by introducing the cowpox virus serpin and caspase-8 inhibitor CrmA in the cells, confirmed that caspase-8 inhibition leads to this form of cell death [4]. Remarkably, while necrosis has been believed in the past to be a passive and accidental form of cell death, it is now considered a finely regulated process [5]. For such a reason it is called necroptosis or programmed necrosis. Necroptosis is characterized by cell swelling, mitochondria dysfunction, plasma membrane permeabilization, and release of cytoplasmic content to the extracellular space. This form of cell death is also associated with high mitochondrial reactive oxygen species (ROS) production and unlike apoptosis it does not involve DNA fragmentation [6].

## 2. Necroptosis Activation and Signalling

Necroptosis can be activated by members of the tumor necrosis factor (TNF) family (through TNFR1, TNFR2, TRAILR1, and TRAILR2), Fas ligand, toll-like receptors, lipopolysaccharides (LPS), and genotoxic stress [2, 7–9]. Also different kinds of physical-chemical stress stimuli can initiate necroptosis, including anticancer drugs, ionizing radiation, photodynamic therapy, glutamate, and calcium overload [10].

Under conditions that are insufficient to trigger apoptosis, TNF*α* activates TNFR1 and in turn induces the recruitment of receptor-interacting protein 1 (RIP1) kinase and other proteins to form complex I. Subsequently, these proteins dissociate from TNFR1 and RIP1 can be found in the cytosol in complex IIb, which includes RIP1, receptor-interacting protein 3 (RIP3) kinase, caspase-8 and FADD. The formation of complex IIb leads to necroptosis [11]. Complex I also includes TRADD which is important in mediating the recruitment of RIP1 kinase to TNFR1 via its death domain (DD) [12].

Necroptosis has been shown to be generally dependent on RIP3, which is activated following phosphorylation by the serine/threonine kinase RIP1 [13]. RIP3 is thought to induce a switch in cell's metabolism, leading to the increase of mitochondrial ROS production that culminates in cell death [14, 15]. The complex RIP1/RIP3 within the so-called necrosome is therefore crucial for the induction of necroptosis. Experiments carried out by multiple experimental approaches have clarified that RIP1 and RIP3 are indeed necessary for necroptosis execution [13, 15, 16]. The RIP1 kinase activity is required for necrosome formation since necrostatin, which allosterically blocks the kinase activity of RIP1, abolishes the assembly of the RIP1-RIP3 complex [13, 16]. While RIP1 involvement has been identified in both apoptosis and necroptosis, RIP3 appears to participate solely in necroptosis. RIP1 and RIP3 have been shown to assemble only in the absence of functional caspase-8, indicating that this enzyme acts as a necrosome inhibitor. Interestingly, caspase-8 has also been shown to cleave, and presumably inactivate, both RIP1 and RIP3 thus acting as a negative regulator of this pathway also through this mechanism. When caspase-8 inactivates RIP1 and RIP3 by proteolytic cleavage, a proapoptotic caspase activation instead of a pronecrotic cascade is triggered [17–19]. Recently the activity of the NAD-dependent deacetylating enzyme SIRT2 has been found to be implicated in the RIP1-mediated recruitment of RIP3 and the necrosome formation [20]. Also the adapter proteins FADD and NEMO appear to be crucial for TNF-alpha-induced necroptosis [21].

The mixed lineage kinase domain like protein (MLKL) has been shown to be an important substrate of RIP3 likely targeting functional downstream targets on cellular organelles such as mitochondria and/or lysosomes [22]. MLKL is phosphorylated by RIP3 at the threonine 357 and serine 358 residues, and these phosphorylation events are critical for necroptosis. In fact blocking MLKL activity leads to necroptosis inhibition. Although the entire molecular mechanism of necroptosis execution is not completely clear these findings implicate MLKL as a key mediator of necroptosis signalling downstream of RIP3 kinase [23].

**Figure 1:**
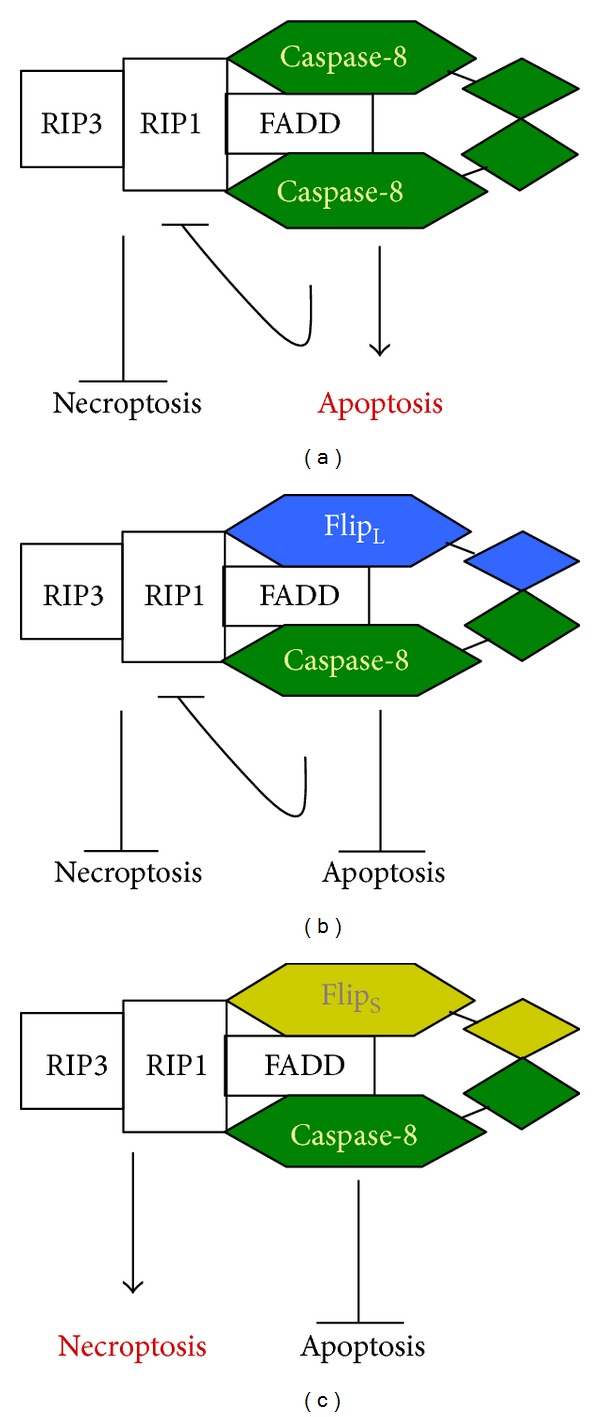
(a) Dimerization of caspase-8 drives apoptosis initiation without triggering necroptosis. (b) c-Flip_L_/caspase-8 heterodimer, by leading to reduced caspase-8 activity, can induce neither apoptosis nor necroptosis. (c) c-Flip_S_/caspase-8 heterodimer, by inhibiting caspase-8, leads to necroptosis induction.

A schematic overview of major signal transduction pathways induced by various stimuli and ultimately leading to necroptosis can be found in Figure 1 of the review article by Kaczmarek et al. [24].

## 3. Flip and Necroptosis

Flip molecules have been originally described as regulators of caspase-8-mediated apoptosis [25] although they are involved in additional functions such as autophagy modulation, proliferative control, cardiac hypertrophy regulation, and Akt/Gsk3*β* activity modulation [26–30]. Different studies indicate that Flip, FADD, and caspase-8 are required for normal embryonic development since their ablation is lethal around E10.5 with similar vascular defects [31–33] suggesting that these proteins display also important nonapoptotic functions during development. Recent evidence indicates that FADD and caspase-8 deficiency can be rescued by RIP3 and RIP1 deletion. Furthermore the lethal effects of Flip deletion are rescued by concurrent ablation of FADD and RIP3. These data suggested that FADD/caspase-8/Flip may negatively regulate RIP1 and RIP3 [34, 35]. More in detail the apoptotic platform constituted by FADD, caspase-8, and Flip has been hypothesized to negatively control necroptosis during development. Although the molecular complex driving necroptosis during development is not completely clear, results obtained *in vitro* confirmed that a caspase-8/Flip heterodimer can inhibit RIP signalling to necroptosis [36]. Furthermore, the embryonic lethality in mice lacking caspase-8, Flip, or the adaptor molecule FADD has been associated with massive necroptosis of endothelial and hematopoietic cells and can be rescued by RIP deletion [37].

Indeed, literature data show that Flip role is quite complex. Flip expression in fact prevents apoptosis that is a caspase-dependent and RIP3-independent cell death. At the same time Flip can inhibit RIP3-dependent necrotic cell death in a caspase-8-dependent manner [36]. In particular caspase-8/Flip heterodimer may prevent the stable association of FADD, RIP1, and RIP3, thereby inhibiting necrotic death. These results support the hypothesis that the main nonapoptotic function of caspase-8 is to suppress RIP3-dependent necrosis during development, likely acting in complex with Flip. The precise mechanism by which the catalytic activity of the caspase-8-Flip complex is engaged to prevent RIP3-dependent necroptosis without triggering apoptosis is not presently known; some additional details are reported below.

## 4. IAPs and Necroptosis

Members of the inhibitors of apoptosis (IAP) protein family are E3 ubiquitin ligases and are well known caspases regulators, characterized by baculoviral IAP repeat domains [38]. During the intrinsic pathway of apoptosis Smac/Diablo is released from mitochondria to cytosol thus removing the inhibition imposed by the IAPs resulting in apoptotic death. Smac protein in fact induces in the cytosol IAP1 and IAP2 autodegradation, allowing the formation of a caspase-8-activating complex containing both RIP1 and caspase-8 [39]. Several mammalian IAPs may utilize ubiquitination to regulate their own stability. It has been recently found that autophagy activation resulted in c-IAP1 and c-IAP2 degradation thus contributing to necroptosis induction [40]. Remarkably, in the absence of IAPs and under conditions where caspases are blocked, necroptosis can be stimulated via RIP1 and its downstream kinase [16]. It has been demonstrated that genotoxic stress or TLR3 stimulation through poly(I:C), a synthetic homologue of virus-derived double stranded DNA, induces IAPs depletion leading to spontaneous aggregation of RIP1 and caspase-8. Such event occurs independently of death receptor stimulation and leads to the formation of “ripoptosome” [41]. The term “ripoptosome” refers to a cell death inducing platform containing RIP1 and most likely RIP3 and regulated by both Flip and IAP proteins (cIAP1, cIAP2, and XIAP). Flip long isoform (Flip_L_) knockdown is able to enhance ripoptosome aggregation thus sensitizing cells to etoposide or TLR3-mediated cell death. IAP proteins are able to inactivate ripoptosome likely inducing proteasomal degradation of RIP1. The role played by ripoptosome is complex since, depending on the cell type, it can stimulate caspase-8-mediated apoptosis or caspase-independent necroptosis [35]. Data from Feoktistova and collaborators demonstrate that in the absence of IAPs (achieved by a IAP antagonist) Flip isoforms levels in the ripoptosome directly control the balance between caspase-dependent apoptosis and RIP-dependent necroptosis [41]. As shown in Figure 1, when Flip proteins are lacking, procaspase-8 homodimers within the ripoptosome lead to caspase-8 activation thus initiating apoptosis. Conversely, in the presence of Flip_L_, caspase-8/Flip_L_ heterodimers may induce RIP cleavage thus leading to ripoptosome disassembly and necroptosis inhibition. Conversely the short Flip isoform (Flip_S_) differently from the long Flip isoform (Flip_L_) promotes RIP3-dependent necroptosis. The caspase-8/Flip_S_ heterodimers lack proteolytic activity necessary for RIP1 degradation thus leading to necroptosis induction via RIP1 and RIP3 [41].

## 5. Necroptosis in Physiology and Pathology

Necroptosis occurs physiologically during development as well as in adult life. Chondrocytes die by necroptosis in human growth plate during bones longitudinal growth [42]. Furthermore, necroptosis may represent an alternative form of death which can substitute apoptosis when caspase activation is blocked. It has been demonstrated that interdigital cells and thymocytes obtained from mice lacking the caspase activator Apaf1 undergo necroptosis instead of apoptosis [43]. Importantly, also in keratinocytes, caspase-8 ablation leads to enhanced necroptosis [44]. It has been hypothesized that an ancestral cell death resembling necrosis was overcome by more recent and more complex processes like autophagy and apoptosis that carry the selective advantage to better contribute to the elimination of cell bodies and organelles [45]. This hypothesis may explain at least in part why, although the ancestral form of cell death is often hidden by other cell death forms, it resumes as a back-up mechanism when the other pathways are blocked.

Necroptosis regulation plays a key role also in the context of immune homeostasis. In fact, whereas the role of apoptosis has been clearly defined in the generation of self-tolerant lymphocytes involved in the establishment of central tolerance, more recently, necroptosis has been implicated in the regulation of T cells proliferation. Previous studies showed that caspase-8, the key molecule mediating apoptosis in response to activation of death receptors, such as Fas [46], also has important nonapoptotic functions [47], as antigen-induced proliferation of T cells required for peripheral T cell homeostasis and T cell survival in response to activation stimuli [48]. In agreement, the specific deletion of caspase-8 in the T cell lineage leads to immunodeficiency associated with impaired T cell homeostasis, T cell lymphopenia, defective T cells proliferation after stimulation with mitogens or antigens, and impaired responses to viral infection [48]. Remarkably, the deficit in T cell expansion caused by loss of caspase-8 was associated with decrease in cell viability but not with apoptosis since no DNA fragmentation was detected. This expansion defect in caspase-8-deficient T cells was rescued by necrostatin or a knockdown of RIP1 [49, 50]. Moreover, it has been later demonstrated that the loss of RIP3 is able to rescue the defective T cell proliferation of casp8−/− mice [36, 49, 51], demonstrating that necroptosis also in T cells is regulated by caspase-8. It is generally accepted that caspase-8 may suppress necroptosis through cleavage and consequent inhibition of RIP1 and RIP3 [17, 19]; it is therefore possible to hypothesize that in physiological conditions caspase-8 is active in suppressing T cells necroptosis, whereas, in pathological conditions, such as viral infection, caspase-8 may be inactivated and consequently T cells may die via necroptosis [49]. Several studies investigated the interaction between key molecules involved in the regulation of necroptosis in T cells. It has been demonstrated that the conditional deletion of FADD, which directly binds to RIP1, leads to impaired lymphocyte proliferation [52–54]. The relationship linking FADD and RIP1 has been more deeply analyzed by Zhang and collaborators [55] demonstrating that levels of RIP1 were increased in FADD−/− embryos in association with necroptosis. By crossing null alleles of RIP1 into FADD−/− mice, normal proliferation of FADD−/− T cells was restored. Moreover, the developmental defect of RIP1−/− lymphocytes was partially corrected by FADD deletion. These data have a dual importance indicating that both apoptosis and necroptosis during T cell development are regulated by the FADD-RIP1 axis. Interestingly, defects in T cells described in FADD−/− mice resemble analogous defects detected in caspase-8−/− mice [31, 32, 56], again confirming the role of caspase-8, and FADD in the control of RIP1-mediated necroptosis in T cells. Another study disclosing the importance of the interplay between apoptotic and necroptotic pathways in T cells has been recently published by Bohgaki et al. [57] demonstrating that the inactivation of caspase-8 in T cells by increasing necroptosis, suppresses autoimmunity caused by Bim deficiency. Bim (Bcl-2-interacting mediator of cell death) is a proapoptotic BH3-only protein belonging to the Bcl-2 protein family, involved in mediating the intrinsic apoptosis pathway [58]. Previous studies demonstrated that the loss of Bim induces autoimmunity due to impaired apoptosis of T cells [59]. Interestingly, Bohgaki and collaborators [57] demonstrated that inactivation of caspase-8 in Bim−/− T cells increases their spontaneous and activation-induced necroptosis thus leading to elimination of Bim−/− T cells. Thus, loss of caspase-8 determines necroptosis of Bim−/− T cells which balances the low apoptotic rate due to Bim deficiency of single mutant Bim−/− T cells. Thus caspase-8 loss in T cells appears to have antagonizing effects on autoimmunity associated with Bim deficiency, suggesting a role for necroptosis in the suppression of autoimmunity. In agreement, the inhibition of the necroptotic process by means of the necroptosis inhibitor necrostatin fully rescued the survival and proliferation of casp8−/− and Bim−/− T cells [57]. Altogether, these data indicate that apoptosis and necroptosis must be tightly regulated in order to maintain immune homeostasis.

Necroptosis has been also associated with different pathological conditions such as ischemia in brain and myocardial tissues, infections [13, 60], neurodegenerative diseases, pancreatitis [16], photoreceptor cell loss [61], and ischaemia-reperfusion damage [62]. Necroptosis may be induced by pathogens (both bacteria and viruses). Recognition of pathogens through pattern-recognition receptors (PRRs) is the first line of defense against infections and some PRRs may initiate necroptosis [24] through RIP1 and/or RIP3 activity [63–65]. Cytomegalovirus infection has been shown to induce RIP3-dependent but RIP1-independent necroptosis thus indicating RIP3 as the main kinase controlling cellular necrotic pathways in such viral pathogenesis [60]. Necroptosis of intestinal epithelial cells seems to be implicated in the pathogenesis of inflammatory bowel diseases. In such pathologies the death receptor 3 (DR3) signalling results in expansion of the Treg pool with concomitant and transient inhibition of Treg suppressive function [66]. It has been demonstrated that mechanisms preventing RIP3-mediated epithelial cell death are crucial for maintaining intestinal homeostasis [67]. Importantly, high levels of RIP3 and increased necroptosis in the ileum of Crohn's disease patients have been found, suggesting a role for necroptosis in the etiopathogenesis of this disease.

There is increasing evidence that necroptosis can be impaired during tumorigenesis. Chronic lymphocytic leukemia cells have been reported to have defects in components involved in necroptosis regulation such as RIP3 and the deubiquitination cylindromatosis (CYLD), an enzyme directly regulating RIP1 ubiquitination [68]. CYLD mutations have been found in epidermal cancer cells [69]. In non-Hodgkin lymphoma, polymorphisms in the RIP3 gene were identified correlating with increased risk to develop the tumor [6]. Necroptosis undoubtedly represents an important process for enhancing tumor cell sensitivity to anticancer treatments and therefore its potentiation may represent an important therapeutic opportunity to kill tumor cells, particularly those resistant to apoptosis. Although apoptotic resistance is a formidable strategy adopted by cancer cells against chemotherapy, cancer cells can be intrinsically susceptible to necroptosis and therefore its induction may represent a valuable tool to counteract their apoptosis resistance [70, 71].

In conclusion necroptosis is emerging as an important process closely interconnected with apoptosis and represents a promising field for innovative therapeutic approaches.
